# Long noncoding RNA ROR regulates chemoresistance in docetaxel-resistant lung adenocarcinoma cells via epithelial mesenchymal transition pathway

**DOI:** 10.18632/oncotarget.16562

**Published:** 2017-03-25

**Authors:** Yan Pan, Jing Chen, Leilei Tao, Kai Zhang, Rui Wang, Xiaoyuan Chu, Longbang Chen

**Affiliations:** ^1^ Department of Medical Oncology, Jinling Hospital, School of Medicine, Nanjing University, Nanjing, Jiangsu, China

**Keywords:** lung adenocarcinoma (LAD), linc-ROR, miR-145, FSCN1, chemoresistance

## Abstract

Emerging evidence indicates that the dysregulation of long non-coding RNAs (lncRNAs) contributes to the development and progression of lung adenocarcinoma (LAD), however the underlying mechanism of action of lncRNAs remains unclear. It is well known that the effective treatment of cancers has been hindered by drug resistance in the clinical setting. Epithelial-mesenchymal transition (EMT) has been recognized to be involved in acquiring drug resistance, cell migration and invasion properties in several types of cancer. Docetaxel-resistant LAD cells established previously in our lab present chemoresistant and mesenchymal features. Long intergenic non-protein coding RNA, regulator of reprogramming (linc-ROR), was first discovered in induced pluripotent stem cells (iPSCs) and was upregulated in docetaxel-resistant LAD cells. In this study, we tried to make clarification of lincRNA-related mechanisms underlying EMT followed by acquired resistance to chemotherapy in LAD. In order to hit the mark, we made use of multiple methods including microarray analysis, qRT-PCR, western blotting analysis, loss/gain-of-function analysis, luciferase assays, drug sensitivity assays, wound-healing assay and invasion assay. We found that decreased expression of linc-ROR effectively reversed EMT in docetaxel-resistant LAD cells and sensitized them to chemotherapy. The function of linc-ROR exerted in LAD cells depended on the sponging of miR-145, therefore, releasing the miR-145 target FSCN1, and thus contributing to the acquisition of chemoresistance and EMT phenotypes of docetaxel-resistant LAD cells. Our findings revealed that linc-ROR might act as potential therapeutic target to overcome chemotherapy resistance in LAD.

## INTRODUCTION

Lung cancer is the disease with the highest morbidity and mortality in malignant tumors [1]. Non-small-cell lung cancer (NSCLC) accounts for about 85% of all lung cancers and among NSCLC, LAD is the most common type [2]. Despite the development of cancer treatment and introduction of new technology, the poor prognosis is still a severe problem in advanced lung cancer. Docetaxel is a chemotherapy medication used to treated many types of cancers, including advanced NSCLC [3]. However, LAD exhibits a high resistance to chemotherapy and recurrence is virtually assured [4]. Emerging evidence indicates a close relationship between chemoresistance and EMT process in cancer [5]. EMT is an important process that plays significant roles in embryonic development, cancers and other diseases [5–7]. This process is characterized by loss of their cell polarity and cell-cell adhesion, and the acquisition of migratory and invasive properties [8]. Therefore, in order to develop novel therapeutic strategies to improve drug resistance, it is extremely important and necessary to determine the mechanisms that connect EMT and the development of drug resistance.

Linc-ROR, firstly reported by Loewer et al in 2010, was described as a 2.6 kb lncRNA and functions in promoting the reprogramming in iPSC [9]. Linc-ROR is located at 18q21.31 and contains the elements of LINE, SINE and LTR [10]. Reports have been confirmed that elevated linc-ROR contributes to the oncogenesis and development of many types of cancers including hepatocellular cancer, breast cancer and endometrial cancer [11–13]. However, linc-ROR and its target genes involved in chemoresistance and EMT are still not fully understood in LAD.

In this study, we identify the function of linc-ROR in regulating chemoresistance and EMT process in LAD cells. Compared with parental control cells (SPC-A1 and H1299), we found out that linc-ROR was upregulated in docetaxel-resistance LAD cells (SPC-A1/DTX and H1299/DTX). Down regulation of linc-ROR reversed the chemoresistance and EMT features of these cells by targeting miR-145 and its target gene FSCN1. Our novel findings provide a potential target for the treatment of chemoresistant LAD patients.

## RESULTS

### linc-ROR is upregulated in docetaxel-resistant LAD cell lines

To identify the possible RNA expression changes in docetaxel resistance in lung adenocarcinoma cells, the mixture A, which was composed of SPC-A1 and H1299 at the same size radio, and the mixture B, which was composed of SPC-A1/DTX and H1299/DTX at the same size radio, were generated. Then a gene chip study was performed in these two mixtures. Compared to mixture A, the expression of linc-ROR was significantly upregulated in mixture B (Supplementary Figure 1D and Supplementary Table 1). QRT-PCR assays were further developed to quantify linc-ROR in two docetaxel-resistant LAD cell lines (SPC-A1/DTX and H1299/DTX) and the corresponding parental cell lines (SPC-A1 and H1299). A significant high expression of linc-ROR was found in docetaxel-resistant LAD cell lines compared with the parental cell lines (Supplementary Figure 1D). These results indicated that linc-ROR might contribute to the chemoresistance phenotype of LAD cells.

### Expression of linc-ROR influences cell proliferation, apoptosis, and docetaxel sensitivity of LAD *in vitro*

**Figure 1:**
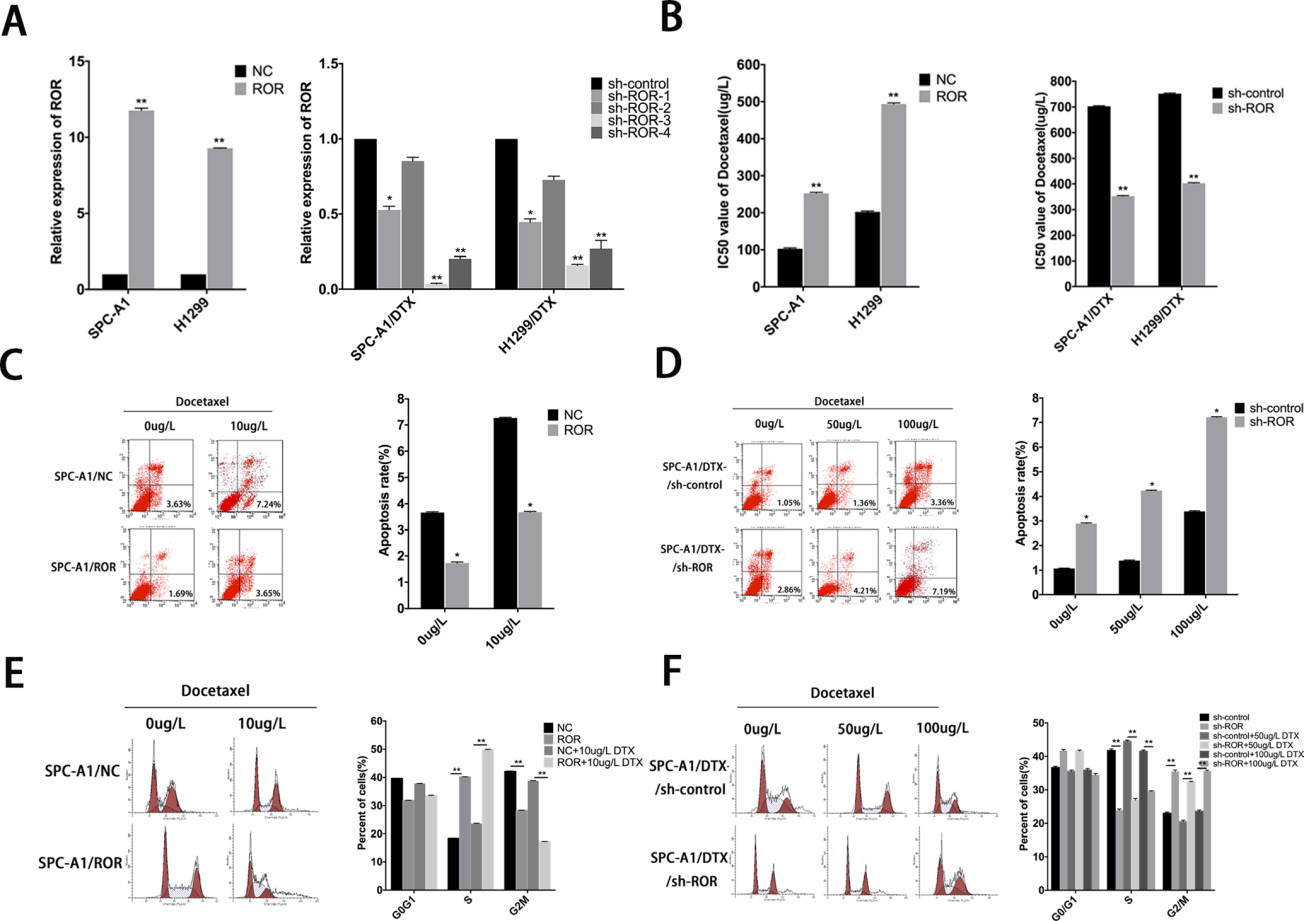
Roles of linc-ROR in chemosensitivity of parental or docetaxel-resistant LAD cells (**A**) qRT-PCR assay was performed to examine the expression of linc-ROR after transfection of SPC-A1 or H1299 cells with linc-ROR (or control) and of SPC-A1/DTX or H1299/DTX cells with sh-ROR-1-4 (or sh-control). (**B**) IC50 values for docetaxel in SPC-A1 and H1299 cells transfected with linc-ROR and SPC-A1/DTX and H1299/DTX cells transfected with sh-ROR. (**C**, **D**) Flow cytometric analysis the influence of linc-ROR on apoptosis rate of SPC-A1/ROR cells or SPC-A1/DTX/sh-ROR cells. (**E**, **F**) Flow cytometric analysis the influence of linc-ROR on the cell cycle of SPC-A1/ROR cells or SPC-A1/DTX/sh-ROR cells. Error bars represent the mean ± SEM of at least three independent experiments. *p < 0.05, **p < 0.01 vs. control group.

To investigate the potential effects of linc-ROR on chemoresistance of LAD, we infected SPC-A1 and H1299 cells with lentivirus to generate cell lines that stably expressed the empty vector and full-length linc-ROR. Meanwhile, SPC-A1/DTX and H1299/DTX cells were infected by lentivirus to generate cell lines that stably knock down of linc-ROR and control cells. QRT-PCR was used to test the efficiency of linc-ROR manipulation. Compared to the control cells, the expression of linc-ROR was significantly upregulated in the stable overexpression cell line and downregulated in the stable knockdown cell lines by four different short hairpin RNA, sh-ROR-3 produced the greatest reduction in endogenous linc-ROR expression (Figure 1A). MTT assays were performed to test the effect of linc-ROR on the IC50 value of docetaxel in LAD cell lines. Compared with control cells, the IC50 value of docetaxel in SPC-A1 / ROR or H1299/ ROR increased significantly (Figure 1B, *p* < 0.01). Conversely, the IC50 value of docetaxel for the SPC-A1/DTX/shROR or H1299/DTX/shROR cells was reduced compared with control cells (Figure 1B, *p* < 0.01). This result demonstrated that the linc-ROR can enhance the resistance of docetaxel in LAD. We obtained similar results from the colony formation assay that the ability to form colonies was significantly enhanced following linc-ROR overexpression in SPC-A1/ROR cells and H1299/ROR cells when exposed to different concentration docetaxel, and greatly decreased in linc-ROR knockdown SPC-A1/DTX/shROR and H1299/DTX/shROR cells to different concentration docetaxel, indicating the function of linc-ROR in proliferation *in vitro* (Supplementary Figure 1A). To further demonstrate the mechanism by which ectopic linc-ROR expression facilitated cell proliferation, we performed flow cytometric analysis of apoptosis and cell cycle. As showed in Figure 1C, 1D and Supplementary Figure 1B, compare with negative controls, after exposure to 0 or 10 μg/L docetaxel for 24 hours, SPC-A1/ROR or H1299/ROR showed stronger resistance to docetaxel-induced apoptosis while SPC-A1/DTX/shROR or H1299/DTX/shROR had high apoptosis rate when exposed to docetaxel (0 μg/L, 50 μg/L, or 100 μg/L, *p* < 0.05). Knockdown of linc-ROR also induces cell percentage increase of G2/M phase, and decreases of S phase in DTX-resistant LAD cells (Figure 1F, Supplementary Figure 1C). Contrarily, overexpression of linc-ROR induces cell percentage decrease of G2/M phase and increase of S phase in parental LAD cells (Figure 1E, Supplementary Figure 1C). Taken together, these data recommended that linc-ROR could enhance the capacity of proliferation and chemotherapy resistance in LAD cells.

### Expression of linc-ROR is associated to the epithelial-mesenchymal transition of docetaxelresistant LAD cells

**Figure 2:**
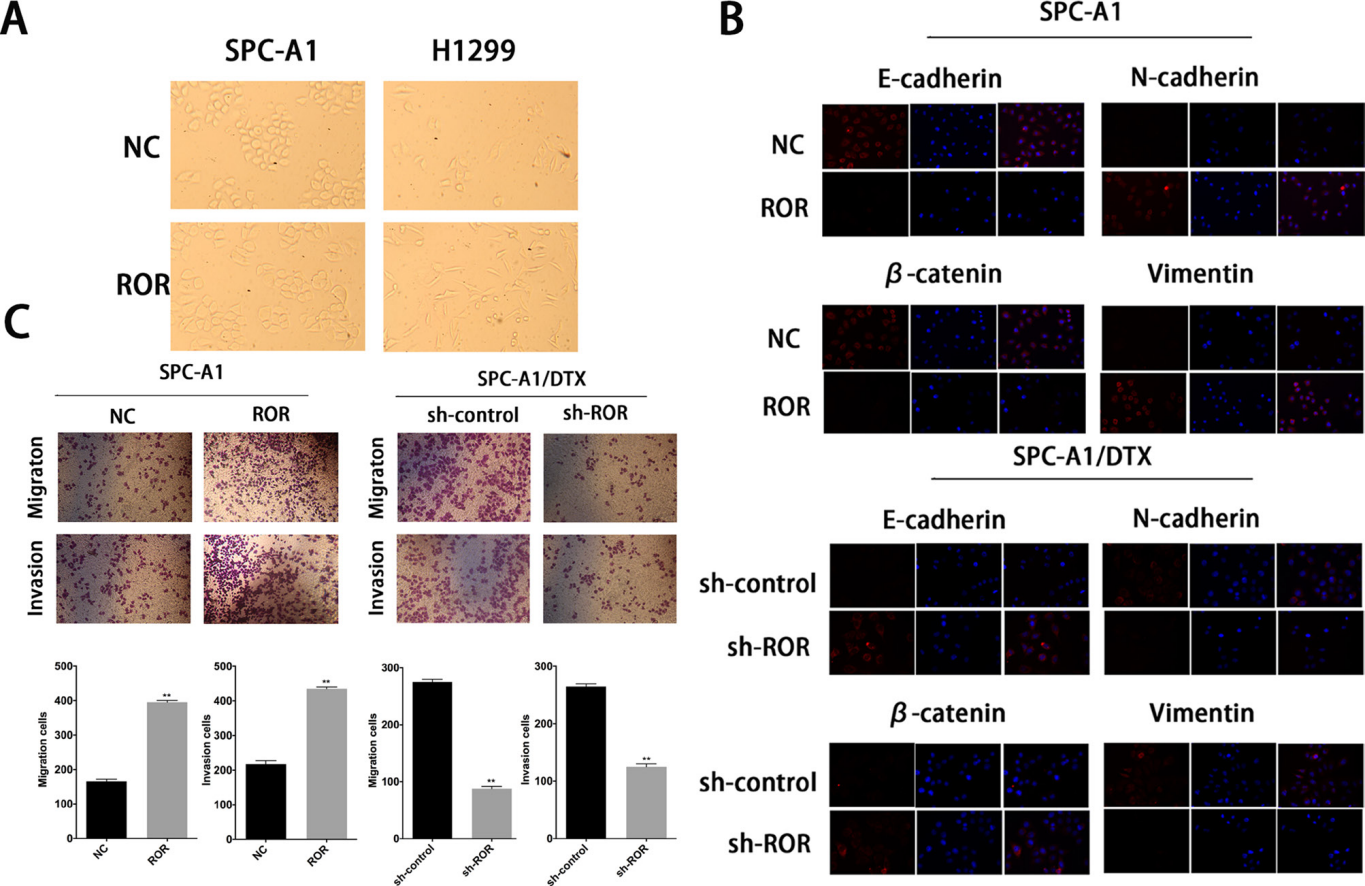
Expression of linc-ROR is associated to the epithelial-mesenchymal transition of docetaxelresistant LAD cells (**A**) The phenotype of SPC-A1/ROR and H1299/ROR cells and their control groups. The SPC-A1/ROR and H1299/ROR cells present a fibroblast-like morphology (typical of mesenchymal phenotype) while the control groups present a round-like morphology (typical of epithelial phenotype). (**B**) Immunofluorescence analysis of the change in epithelial and mesenchymal markers in SPC-A1/ROR cells and in SPC-A1/DTX/sh-ROR cells. (**C**) Metastasis capacity of SPC-A1/ROR cells and SPC-A1/DTX/sh-ROR cells by transwell assays. Error bars represent the mean ± SEM of at least three independent experiments. *p < 0.05, **p < 0.01 vs. control group.

EMT process confers invasive capacity, apoptosis, and drug resistance to the transformed epithelial cells [14]. As shown in Figure 2A, upregulation of linc-ROR in SPC-A1 and H1299 cells leaded to a fibroblast-like morphology, which is typical of the mesenchymal phenotype of cells associated with the loss of epithelial markers compared with the corresponding control groups. To identify whether silencing of linc-ROR could abolish the invasiveness and metastasis of lung cancer cells via going through abolishing the EMT process, we detected the biomarkers of EMT by western blotting and immunofluorescent staining in SPC-A1 (or H1299) and SPC-A1/DTX (or H1299/DTX) cells in response to different levels of linc-ROR. As shown in Supplementary Figure 2A, forced expression of linc-ROR reduced the expression of E-cadherin and β-catenin, which are the characteristic biomarkers of epithelial cells, and increased the expression of N-cadherin and Vimentin, indicating the display of mesenchymal phenotype. Conversely, downregulation of linc-ROR increased the levels of epithelial markers and decreased the levels of mesenchymal markers (Supplementary Figure 2A). Moreover, results obtained from immunofluorescence studies showed a similar change in marker expression (Figure 2B and Supplementary Figure 2B). Furthermore, both transwell and “wound healing” assays showed increased invasion and migration activity of parental LAD cells in the presence of linc-ROR, but remarkably reduced invasion and migration activity in the absence of linc-ROR in SPC-A1/DTX (or H1299/DTX) cells (Figure 2C and Supplementary Figure 3A, 3B). These observations led to the hypothesis that the expression of linc-ROR may be correlated with the EMT in tumor cells.

### Inhibition of linc-ROR sensitizes LAD to DTX *in vivo*

**Figure 3:**
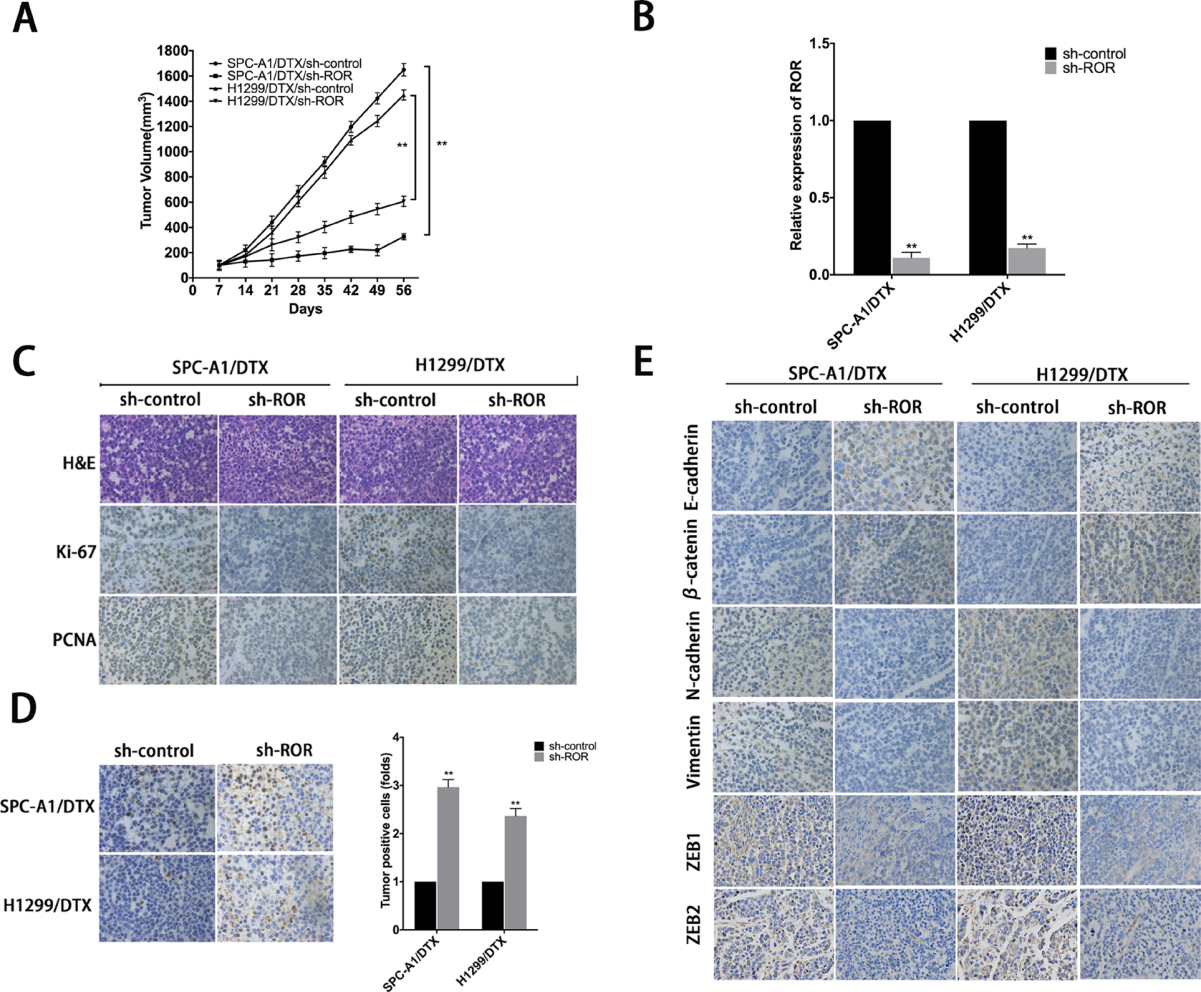
Effect of linc-ROR on chemoresistance and EMT of docetaxel-resistant LAD cells *in vivo* (**A**) The xenograft tumors were obtained and measured and the values of tumor volume after transplantation. (**B**) The relative expression of linc-ROR in four groups. (**C**) The immunohistochemistry staining of ki-67 and proliferating cell nuclear antigen (PCNA) in tumor samples. (**D**) TUNEL staining in tumor samples. (**E**) The immunohistochemistry staining of epithelial markers (E-cadherin, β-catenin) and mesenchymal markers (N-cadherin, vimentin) and ZEB1 and ZEB2. Error bars represent the mean±SEM of at least three independent experiments. *p < 0.05, **p < 0.01 vs. control group.

To investigate the effect of linc-ROR knockdown on DTX-resistant LAD cells *in vivo*, SPC-A1/DTX and H1299/DTX cells with stable transfection of sh-ROR were subcutaneously transplanted into nude mice. Two weeks later all tumors were separated after the treatment with 1 mg/kg DTX, which were intraperitoneally injected into the nude mice. With the treatment of DTX, the tumor volume formatted by SPC-A1/DTX and H1299/DTX cells transfected with sh-ROR was lighter or smaller than control (Figure 3A, Supplementary Figure 1E). QRT-PCR was performed to test the expression of linc-ROR in four groups, results shows that groups transfected with sh-ROR have significantly lower levels of linc-ROR compared to control groups (Figure 3B). Hematoxylin and eosin staining was performed to confirm xenograft tumor morphology, and immunohistochemical staining was performed to show the decreased positive rates of proliferation markers ki-67 and proliferating cell nuclear antigen (PCNA) (Figure 3C). Terminal deoxynucleotidyl transferase-mediated nick end labeling (TUNEL) staining showed more cell apoptosis in tumors with linc-ROR knockdown (Figure 3D). Furthermore, we also performed immunohistochemical to detect the expression of epithelial and mesenchymal markers and two important actors of EMT, ZEB1 and ZEB2, in subcutaneous tumors formed from cells transfected with sh-ROR. As shown in Figure 3E, the levels of epithelial protein markers were increased, while those of mesenchymal markers, ZEB1 and ZEB2 were decreased, compared with control groups. These results indicated that the downregulation of linc-ROR sensitizes docetaxel-resistant LAD cells to docetaxel treatment and abolishes the invasiveness and metastasis of them.

### Linc-ROR regulates expression of miR-145 to induce resistance of LAD cells to DTX and EMT

**Figure 4:**
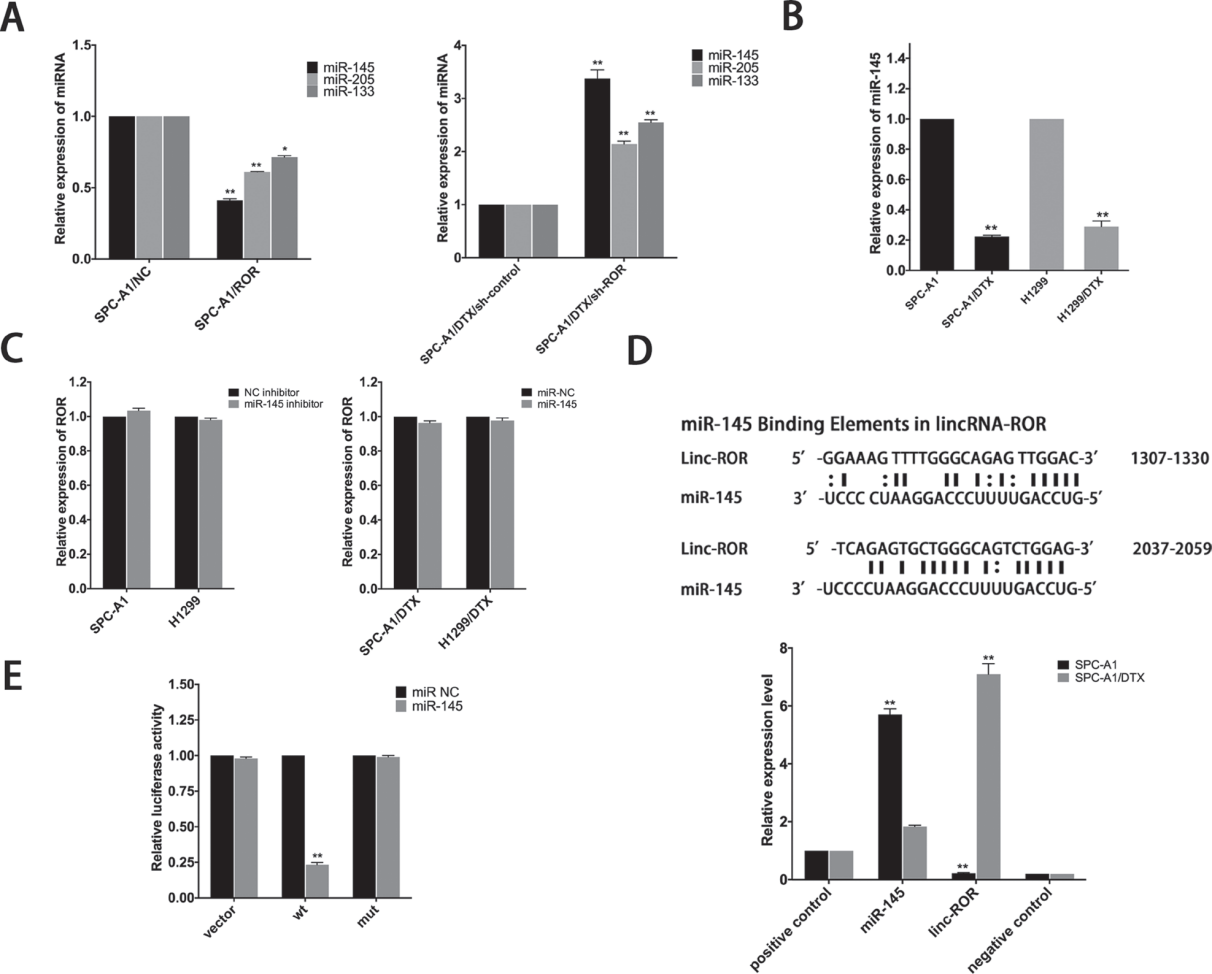
MiR-145 is a target of linc-ROR (**A**) Effect of dysregulated linc-ROR expression on the level of miR-145, miR-205 and miR-133 was measured by qRT-PCR. (**B**) QRT-PCR analysis of the expression level of miR-145 in parental and docetaxel-resistant LAD cells. (**C**) qRT-PCR was employed to detect the effect on linc-ROR level after ectopic expression or knockdown of miR-145. (**D**) Bioinformatics predicted miR-145 binding sites at two distinct positions in linc-ROR. RIP assay to detect the association between linc-ROR and miR-145 in SPC-A1 and SPC-A1/DTX cells. The positive and negative controls refer to U1 and IgG, respectively. (**E**) Luciferase activity in SPC-A1 cells co-transfected with miR-145 and luciferase reporters containing no insert, linc-ROR, or mutant linc-ROR. Data are presented as the relative ratio of firefly luciferase activity to Renilla luciferase activity. Error bars represent the mean ± SEM of at least three independent experiments. *p < 0.05, **p < 0.01 vs. control group.

Reports have suggested that linc-ROR can function as a competing endogenous RNA (ceRNA) and overexpression of it may reverse the negative regulation between miRNAs and their target genes. Related miRNAs consist of miR-145, miR-205 and miR-133 [11, 13, 15–19]. In this study, qRT-PCR was performed to compare the expression of miR-145, miR-205 and miR-133 in linc-ROR downregulated and upregulated LAD cells, results showed that all of them can be regulated by linc-ROR, especially miR-145 (Figure 4A). We also tested the expression of miR-145 in LAD cells by qRT-PCR, as shown in Figure 4B, expression of miR-145 sharply decreased in docetaxel-resistant LAD cells. Additionally, there was no difference in linc-ROR levels after ectopic expression or knockdown of miR-145 (Figure 4C). To further validate the regulatory relationship between linc-ROR and miR-145, we performed an RNA immunoprecipitation (RIP) assay that revealed a competitive relationship between linc-ROR and miR-145 (Figure 4D). The results of luciferase reporter assays provided further confirmation. As shown in Figure 4E, miR-145 mimics reduced the luciferase activity of wild-type (WT) linc-ROR reporter vector but not that of empty vector and mutant reporter vector. The results suggested miR-145 as a direct downstream target of linc-ROR.

### MiR-145 mediates drug resistance and EMT phenotype of LAD cells *in vitro*

**Figure 5:**
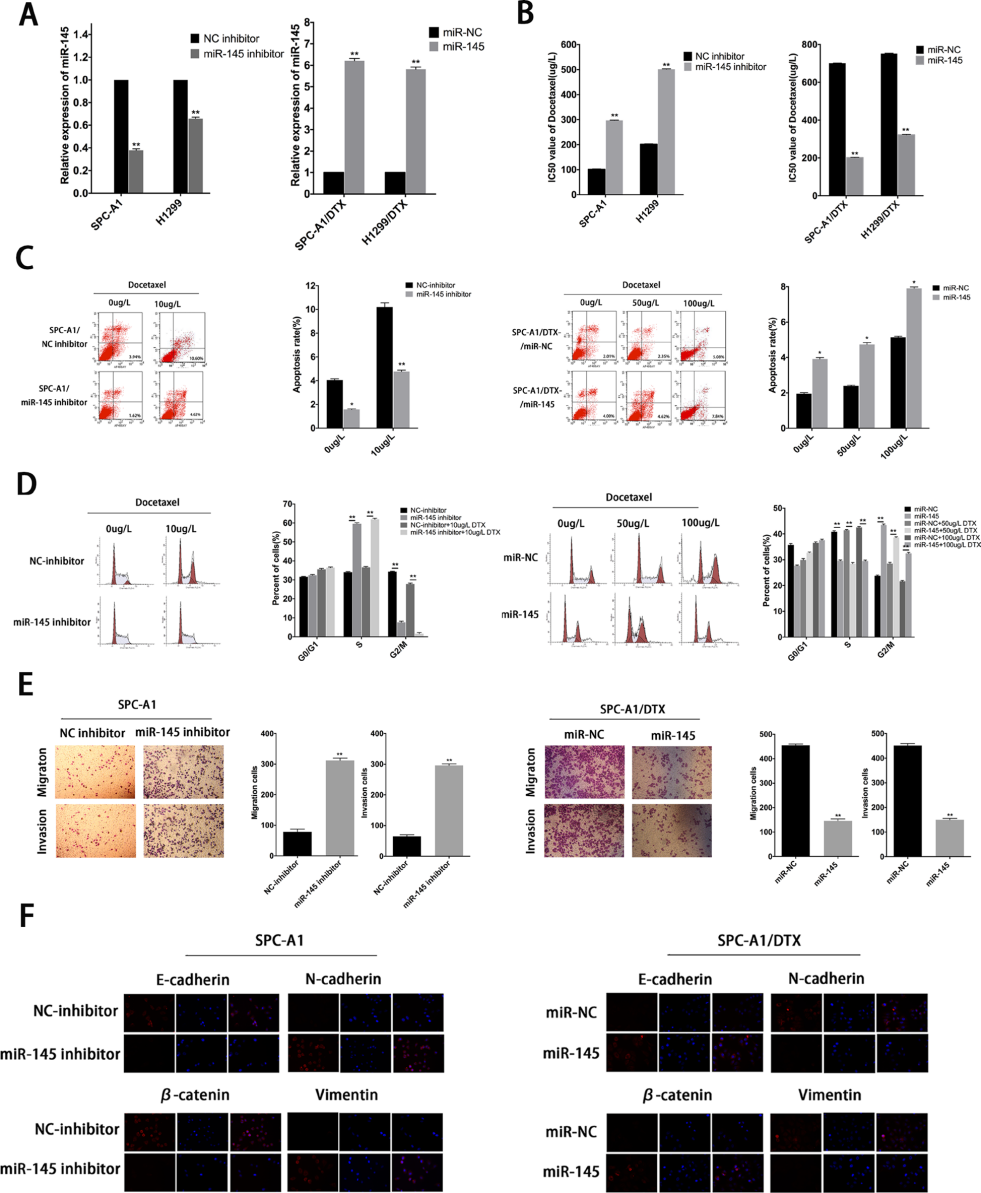
MiR-145 mediates drug resistance and EMT phenotype of LAD cells *in vitro* (**A**) QRT-PCR assay was performed to examine the expression of miR-145 after transfection of SPC-A1 or H1299 cells with miR-145 inhibitor (or NC inhibitor) and of SPC-A1/DTX or H1299/DTX cells with miR-145 mimics (or miR-NC). (**B**) IC50 values for docetaxel in SPC-A1 and H1299 cells transfected with miR-145 inhibitor and SPC-A1/DTX and H1299/DTX cells transfected with miR-145 mimics. (**C**) Flow cytometric analysis the influence of miR-145 on apoptosis rate of SPC-A1 cells or SPC-A1/DTX cells. (**D**) Flow cytometric analysis the influence of miR-145 on cell cycle of SPC-A1 cells or SPC-A1/DTX cells. (**E**) Metastasis capacity of miR-145 inhibitor-transfected SPC-A1 cells or miR-145 mimics-transfected SPC-A1/DTX cells by transwell assays. (**F**) Immunofluorescence analysis of the change in epithelial and mesenchymal markers in miR-145 inhibitor-transfected SPC-A1 cells or miR-145 mimics-transfected SPC-A1/DTX cells. Error bars represent the mean ± SEM of at least three independent experiments. *p < 0.05, **p < 0.01 vs. control group.

EMT can modulate cancer progression and metastasis and is also implicated in the onset of drug resistance. To determine whether miR-145 was involved in drug resistance and EMT phenotype, we transfected miR-NC or miR-145 mimics into SPC-A1/DTX (or H1299/DTX cells) and NC inhibitor or miR-145 inhibitor into SPC-A1 (or H1299) cells. The transfection efficiency was satisfied about 48 hours after transfection (Figure 5A). The IC50 values of DTX in miR-145 overexpressed DTX-resistant cells are lower than control (Figure 5B). On the other hand, the IC50 value for DTX in SPC-A1 (or H1299) cells transfected with miR-145 inhibitor was higher than control, respectively (Figure 5B). Colony formation assay showed that the proliferation ability of DTX-resistant cells with overexpressed miR-145 diminished significantly and the opposite effect for SPC-A1 (or H1299) transfected with miR-145 inhibitor (Supplementary Figure 1A). Moreover, overexpression of miR-145 induced more cell apoptosis and caused increase of S phase and G2/M phase arrest in cell cycle distribution (Figure 5C, 5D and Supplementary Figure 1B, 1C). Additionally, western blot analysis and immunofluorescence studies were performed to investigate the function of miR-145 on the induction of EMT in LAD cells. The expression of epithelial markers was decreased while that of mesenchymal markers was increased following transfection of miR-145 inhibitor into SPC-A1 (or H1299 cells) (Figure 5F and Supplementary Figure 2A, 2B). The opposite results were obtained for SPC-A1/DTX (or H1299/DTX) transfected with miR-145 mimics (Figure 5F and Supplementary Figure 2A, 2B). Furthermore, both transwell invasion and “wound healing” assays showed a significant increase in the invasive capacity of SPC-A1 (or H1299) cells in the presence of miR-145 inhibitor (Figure 5E and Supplementary Figure 3A, 3B). In contrast, the opposite results were obtained for SPC-A1/DTX (or H1299/DTX) cells treated with miR-145 mimics (Figure 5E and Supplementary Figure 3A, 3B).

All these results strongly suggest that limited expression of miR-145 might contribute to the therapeutic resistance and EMT transition, thereby promoting the oncogenic function of linc-ROR in LAD cells.

### The oncogenic function of linc-ROR in LAD cells *in vitro* is dependent on miR-145

**Figure 6:**
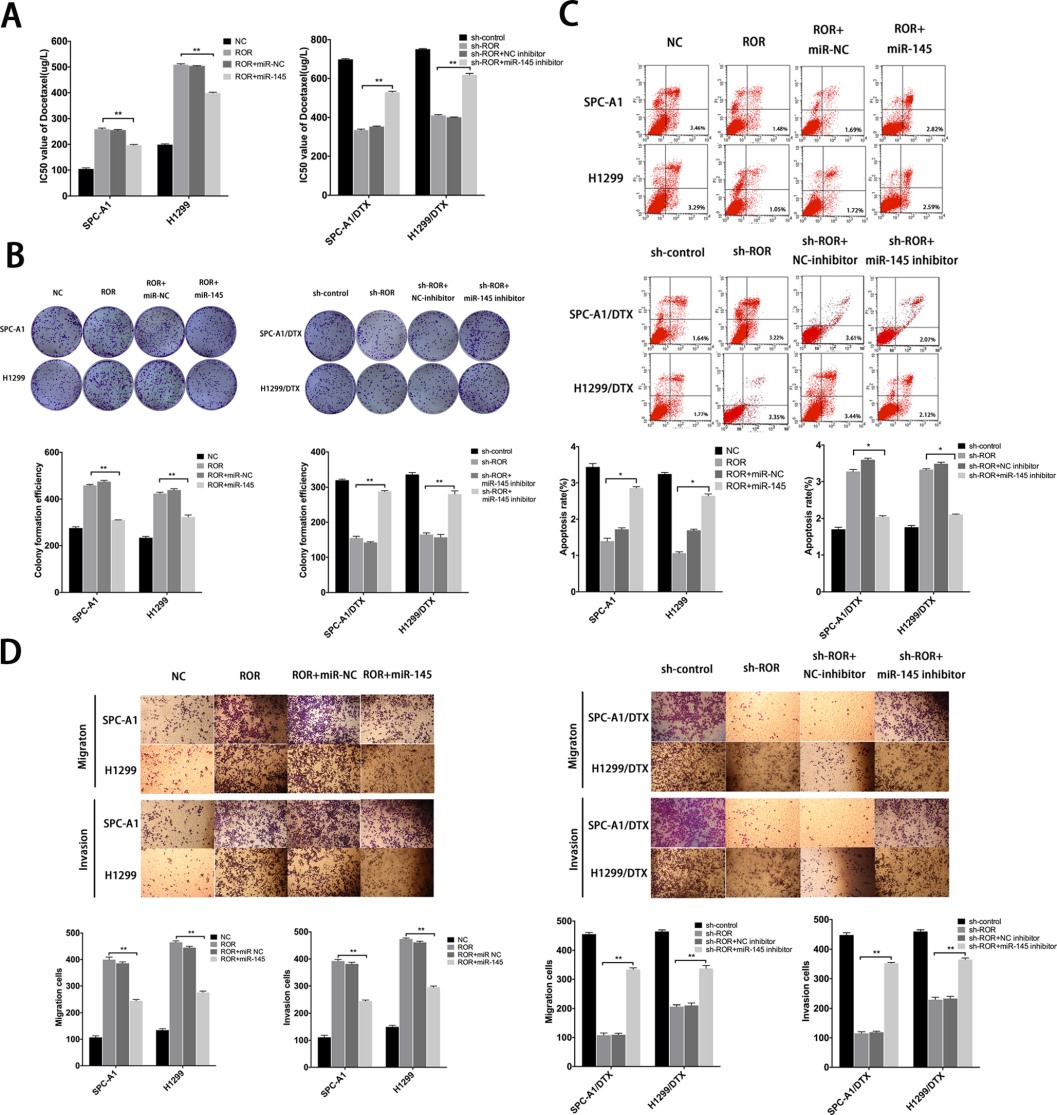
Function of linc-ROR in chemoresistance of LAD cells is partially reversed by miR-145 (**A**) IC50 values of SPC-A1 and H1299 cells stably transfected with linc-ROR and SPC-A1/DTX and H1299/DTX cells stably transfected with sh-ROR was partially reversed by miR-145 mimics and miR-145 inhibitor, respectively. (**B**) Colony formation assays to determine the influence of miR-145 on linc-ROR. (**C**) Apoptosis rates of LAD cells lines were detected to determine the influence of miR-145 on linc-ROR. (**D**) Metastasis capacity of SPC-A1 and H1299 cells stably transfected with linc-ROR and SPC-A1/DTX and H1299/DTX cells stably transfected with sh-ROR was partially reversed by miR-145 mimics and miR-145 inhibitor, respectively. Error bars represent the mean ± SEM of at least three independent experiments. *p < 0.05, **p < 0.01 vs. control group.

To investigate whether linc-ROR influenced LAD cell proliferation, apoptosis, and EMT process were depended on miR-145, we performed rescue experiments including MTT assays, colony formation assays, flow cytometry assays, wound healing assays and transwell assays. MiR-NC or miR-145 inhibitor were transfected into SPC-A1/DTX (or H1299/DTX) cells stably transfected with sh-control or sh-ROR, and miR-NC or miR-145 mimics were transfected into SPC-A1 (or H1299) cells stably transfected with linc-ROR. Cotransfection could partially rescue promotion of colony formation capacity and the decreased chemosensitivity of docetaxelresistant LAD cells induced by linc-ROR (Figure 6A, 6B). Furthermore, antiapoptotic effect of linc-ROR could be partially reversed by the introduction of miR-145 mimics into SPC-A1 (or H1299) cells treated with docetaxel, and a similar antiapoptotic effect after exposure to docetaxel (0 μg/L, 50 μg/L, or 100 μg/L) was also observed after co-transfection of sh-ROR and miR-145 inhibitors into docetaxel-resistant LAD cells (Figure 6C). Additionally, transwells assays and “wound healing” assays showed that the pro-metastasis and invasive effect of linc-ROR in SPC-A1 (or H1299) cells could be partly abolished by cotransfection with miR-145 mimics, whereas the anti-metastasis effect induced by sh-ROR in SPC-A1/DTX (or H1299/ DTX) cells could be partially reversed by miR-145 inhibitors (Figure 6D and Supplementary Figure 3C). Taken together, restoration of miR-145 can partially rescue the phenotypic changes, including proliferation, apoptosis, docetaxel chemosensitivity, and the induction of the EMT phenotype, in docetaxel-transfected LAD cells, which were induced by linc-ROR, further suggesting that linc-ROR functions in docetaxel-resistant LAD cells, at least by part, by downregulation of miR-145.

### FSCN1 is a functional target of miR-145

**Figure 7:**
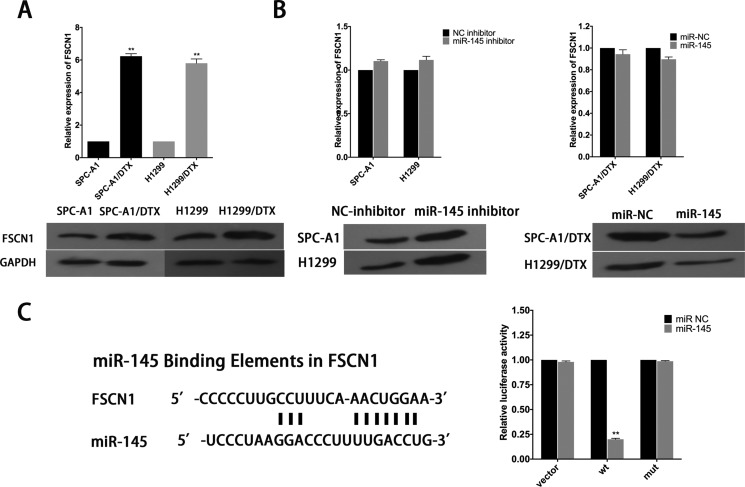
FSCN1 is a target of miR-145 (**A**) qRT-PCR and western blot analysis of the expression level of FSCN1 in parental and docetaxel-resistant LAD cells. (**B**) Effect of dysregulated miR-145 expression on the level of FSCN1 was measured by qRT-PCR and western blot analysis. (**C**) Bioinformatics predicted miR-145 binding sites at FSCN1. Luciferase activity in SPC-A1/DTX cells co-transfected with miR-145 and luciferase reporters containing no insert, FSCN1, or mutant FSCN1. Data are presented as the relative ratio of firefly luciferase activity to Renilla luciferase activity. Error bars represent the mean ± SEM of at least three independent experiments. *p < 0.05, **p < 0.01 vs. control group.

**Figure 8:**
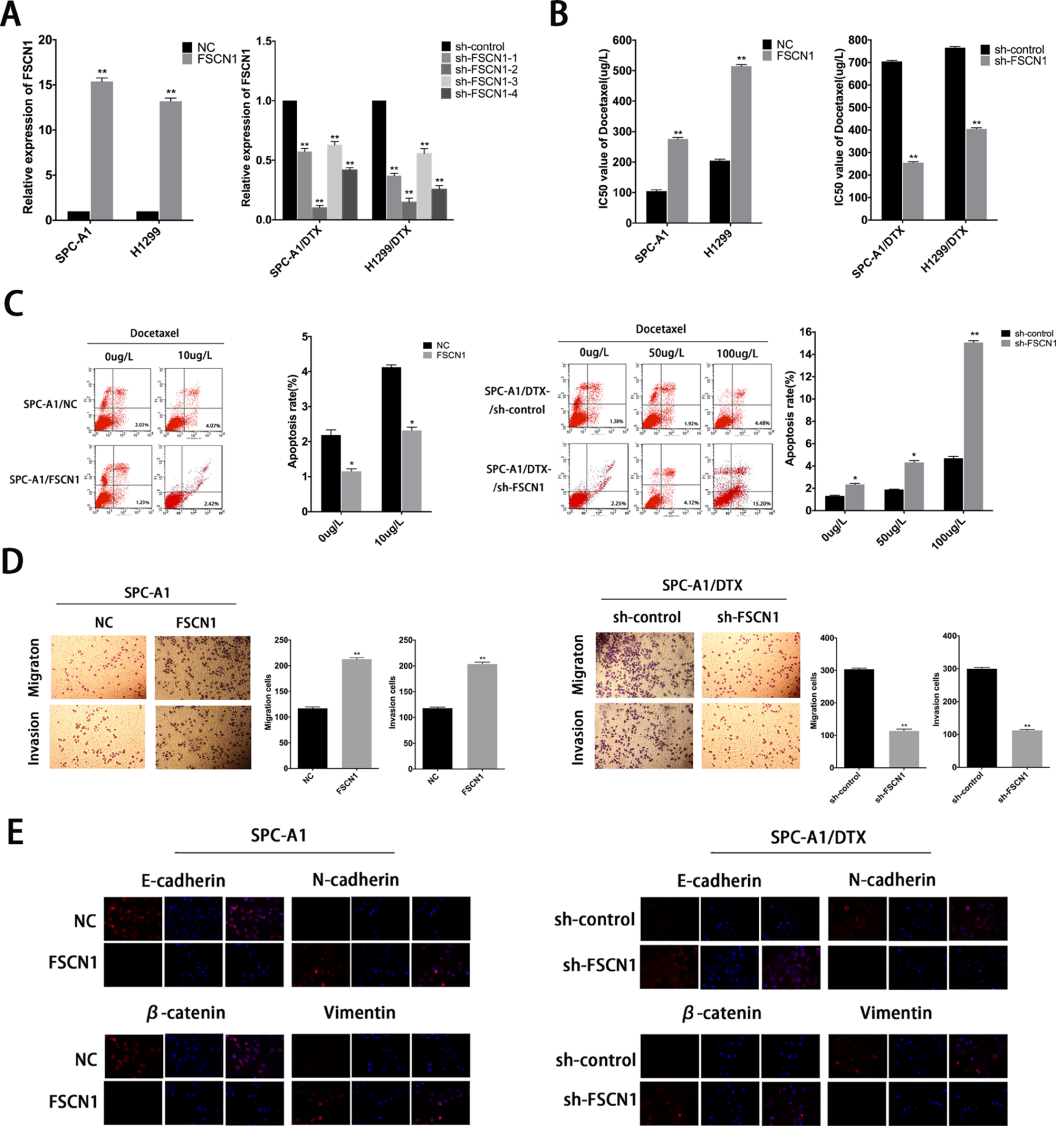
Roles of FSCN1 in chemoresistance and EMT process of docetaxel-resistant LAD cells (**A**) QRT-PCR assay was performed to examine the expression of FSCN1 after transfection of SPC-A1 or H1299 cells with FSCN1 (or control) and of SPC-A1/DTX or H1299/DTX cells with sh-FSCN1-1-4 (or sh-control). (**B**) IC50 values for docetaxel in SPC-A1 and H1299 cells transfected with FSCN1 and SPC-A1/DTX and H1299/DTX cells transfected with sh-FSCN1. (**C**) Flow cytometric analysis the influence of FSCN1 on apoptosis rate of SPC-A1 cells or SPC-A1/DTX cells. (**D**) Metastasis capacity of FSCN1-transfected SPC-A1 cells or sh-FSCN1-transfected SPC-A1/DTX cells by transwell assays. (**E**) Immunofluorescence analysis of the change in epithelial and mesenchymal markers in FSCN1-transfected SPC-A1 cells or sh-FSCN1-transfected SPC-A1/DTX cells. Error bars represent the mean±SEM of at least three independent experiments. *p < 0.05, **p < 0.01 vs. control group.

Potential targets of miR-145 were analyzed and chosen by employing open access online miRNA target databases. Fascins (FSCNs) are 55-kDa globular proteins composed of four tandem FSCN domains, each of which corresponds structurally to a β-trefoil fold [20]. FSCN1, a member of the FSCN family of actin-binding proteins, has an important role in cell proliferation, migration and invasion in various types of cancer and was identified as a predictable downstream target of miR-145 [21, 22]. FSCN1 expression was tested in SPC-A1 and SPC-A1/DTX cells. The results showed that both mRNA and protein level of FSCN1 are higher in SPC-A1/DTX than in SPC-A1 (Figure 7A). To examine the inhibitory effect of miR-145 on FSCN1 mRNA and protein levels, we conducted qRT-PCR assay and Western blotting at 48 hours after transfected with miR-145 inhibitor or miR-145 mimics into SPC-A1/DTX (or H1299/DTX) or SPC-A1 (or H1299) cells. Overexpression of miR-145 significantly decreased the expression level of FSCN1 protein in docetaxel-resistant LAD cells, whereas downregulation of miR145 led to the increased expression level of FSCN1 protein in parental cells, but miR-145 has no influence on the mRNA levels of FSCN1 (Figure 7B). Then, as shown in Figure 8C, the 3′-UTR of FSCN1 is complementary binding site for miR-145. Luciferase reporter containing amplified FSCN1 3′-UTR segment with or without mutated the miR-145 potential binding site were respectively transfected into SPCA1/DTX cells overexpressed miR-145. The results show that overexpression of miR-145 could inhibit the activity of luciferase that carried wild-type but not mutant 3′-UTR of FSCN1 (Figure 7C). Thus, miR-145 might negatively regulate the expression of FSCN1 in docetaxel-resistant LAD cells by post-translational control.

### Roles of FSCN1 in formation of chemoresistance and EMT phenotype in docetaxel-resistant LAD cells induced by downregulation of miR-145

To explore the biologic functions of FSCN1 in docetaxel-resistant LAD cells, 4 siRNAs (sh-FSCN1-1-4) were used to knockdown endogenous FSCN1 expression, we chose sh-FSCN1-2 for the following experiments, and FSCN1 plasmid was used to upregulate the expression of FSCN1 in parental cells (Figure 8A). FSCN1 downregulation could lead to the decreased IC50 values of SPC-A1/DTX or H1299/DTX cells, respectively (Figure 8B). On the other hand, IC50 value for docetaxel in SPC-A1 (or H1299) cells transfected with FSNC1 was increased compared with NC group (Figure 8B). Also, FSCN1 downregulation resulted in the decreased colony formation capacity of docetaxel-resistant LAD cells whereas upregulation of FSCN1 leads to the increased colony capacity of parental LAD cells (Supplementary Figure 1A). Moreover, overexpression of FSCN1 induced less cell apoptosis while downregulation of FSCN1 leads to more cell apoptosis (Figure 8C and Supplementary Figure 1B). The effects of FSCN1 on expression of EMT-related molecular markers were then determined by western blot analysis and immunofluorescence studies. Forced expression of FSCN1 reduced the expression of epithelial markers and increased the expression of mesenchymal markers (Figure 8E and Supplementary Figure 2A, 2B). Conversely, downregulation of FSCN1 increased the levels of epithelial markers and decreased the levels of mesenchymal markers (Figure 8E and Supplementary Figure 2A, 2B). Cell migration/invasion assays revealed a facilitating effect of FSCN1 on metastasis of parental LAD cells (Figure 8D and Supplementary Figure 3A, 3B). In contrast, SPC-A1/DTX (or H1299/DTX) cells transfected with sh-FSCN1 showed relatively low migration and invasion capability compared with negative control groups (Figure 8D and Supplementary Figure 3A, 3B). Generally, we demonstrated negative regulation in miR-145 by linc-ROR, which contributes to chemoresistance and EMT phenotype of LAD by regulating FSCN1 expression.

## DISCUSSION

Accumulating evidence suggests that the majority of lncRNAs are biologically relevant [23]. And dysregulation of lncRNAs has also been shown to contribute to cancer pathogenesis, providing novel therapeutic opportunities to treat cancer [24]. However, the roles of lncRNAs in LAD carcinogenesis are not well understood. Chemoresistance is one of the main obstacles in the clinical treatment of patients diagnosed with LAD. Moreover, the emergence of EMT contributes to the malignancy of LAD. Therefore, investigating the mechanisms underlying chemoresistance and the EMT phenotype in LAD might facilitate the development of novel treatments that improve the patient prognosis.

It is reported that EMT process plays a vital role in promoting drug resistance, recurrence and metastasis during the course of tumor treatment [25]. So far, few reports have revealed that EMT transition also contributes to therapeutic resistance in lung cancer. Sato H et al. hypothesized that the miR-200c might serve as a therapeutic target for EMT-related EGFR-TKI resistance in lung cancer [26]. Ye and his colleagues reported that the EMT process could be inhibited by the restoration of miR-101 in NSCLC cells, finally leading to the increasing sensitivity to cisplatin in lung cancer [27]. Also, it's reported that miR-181a is associated with EMT progression and chemoresistance in lung adenocarcinoma, potentially through targeting of PTEN [28]. In line with these reports, in the present study, we showed that there is a similarity between morphological characteristics of docetaxel-resistant cells and EMT features, which include the decreased epithelial markers (E-cadherin and β-catenin) and increased mesenchymal markers (N-cadherin and Vimentin) [29]. However, the molecular mechanisms accounting for acquisition of EMT phenotype in docetaxel-resistant LAD cells remain largely unclear and need to be further elucidated.

NcRNAs, including miRNAs, have been shown to promote tumorigenesis [30, 31]. Several studies have implicated the involvement of lncRNA in neurological disease and oncogenesis [32]. It is suggested that lncRNA may act as ceRNA to suppress the biological functions of miRNAs [33–35]. Similar to those reports, overexpression of linc-ROR can reverse the negative regulation between miRNAs and their target genes [36]. Linc-ROR is reported to stimulate EMT process by acting as a ceRNA for miR-205 in breast cancer cells [16]. Furthermore, Wang et al. found that linc-ROR enhanced the expression of Oct4, Sox2 and Nanog by sponging the differentiation-related miRNAs in hESCs especially miR-145 [9, 15]. Although linc-ROR has been reported in a few types of cancers, but the roles of it in LAD carcinogenesis are still not understood. In our study, we compared the expression of miR-145, miR-205 and miR-133 in linc-ROR downregulated and upregulated LAD cells, results showed that miR-145 changed most among three miRNAs. MiR-145 is hypothesized to be a tumor suppressor and is down-regulated in various types of cancers, including breast cancer and lung cancer [37, 38]. Therefore we hypothesized that linc-ROR might function in LAD in a miR-145-dependent manner. To test this hypothesis, we performed RIP and luciferase reporter assays to confirm the association between linc-ROR and miR-145. Then, basing on our several experimental results, we further confirmed the importance of linc-ROR and miR-145 in mediating chemotherapy resistance and EMT phenotype in LAD cells. Also, potential targets of miR-145 were analyzed and chosen by employing open access online miRNA target databases. FSCN1 was identified as a predictable downstream target of miR-145. It is reported that FSCN1 protein can locate in the plasma membrane and thus promotes cell motility [39]. It has also been well established that FSCN1 can increase the capacities of cellular interactions, cell adhesion motility and proliferation [20, 40]. There is evidence that miR-145 could inhibit tumor cell proliferation, invasion and metastasis by targeting FSCN1 [41–45]. In our study, we also confirmed that FSCN1 functions as a miR-145 target and it contributes to the chemoresistance and EMT phenotype in docetaxel-resistant LAD cells.

Taken together, we first identified that dysregulation of linc-ROR/miR-145/FSCN1 signalling pathway was associated with therapeutic resistance and EMT transition in LAD cells. In our study, the inhibited expression of linc-ROR could not only increase sensitivity to docetaxel and reverse EMT process, but also reduce the capacity of proliferation, migration and invasion in docetaxel-resistant LAD cells. MiR-145 was identified as a direct target of linc-ROR, and overexpression of miR-145 would lead to the downregulation of its target gene FSCN1. This newly identified linc-ROR/miR-145/FSCN1 axis provides potential therapeutic strategies for drug resistance in LAD patients.

## MATERIALS AND METHODS

### Cell lines

Two human LAD cell lines SPC-A1 and H1299 were purchased from the Tumor Cell Bank of Chinese Academy of Medical Science (Shanghai, China) and cultured in RPMI 1640 medium containing 10% fetal bovine serum and ampicillin and streptomycin at 37°C in a humidified atmosphere of 95% air and 5% CO2. The docetaxel-resistant LAD cells (SPC-A1/DTX and H1299/DTX) derived from parental SPC-A1 and H1299 cells, respectively, were established and preserved in 50 ug/L final concentration of docetaxel.

### LncRNA microarray analysis

LncRNA microarray analysis was done in the parental mixture A (SPC-A1 and H1299) and doxcetaxel-resistant cell lines mixture B (SPC-A1/DTX and H1299/DTX). Briefly, total RNA was isolated using RNAiso Plus (TAKARA, Japan) and the RNeasy mini kit (Qiagen) according to the manufacturer's instructions. Depending on the Agilent Human lncRNA 4 * 180 genechip from Shanghai Biotechnology Corporation, linc-ROR was selected, whose expression levels between the mixture A and mixture B differed by 9.05-fold.

### Real-time quantitative reverse-transcription polymerase chain reaction (qRT-PCR)

Total RNA from tissues and cells was isolated with Trizol reagent (Invitrogen, CA, USA) according to the manufacturer's instructions. Reverse transcription was performed with PrimeScript RT reagent Kit (Takara, Japan) according to the manufacturer's protocol. qRT-PCR was performed with SYBR Prime Script RT-PCR Kits (Takara, Japan) based on the manufacturer's instructions. The linc-ROR, miR-145 and FSCN1 level was calculated with the 2^ΔΔCt^ method, which were normalized to GAPDH mRNA or U6 rRNA, respectively. All assays were performed in triplicate. The expression levels were relative to the fold change of the corresponding controls which were defined as 1.0. PCR primers were designed as follows:

lincROR forward:5′- GAATCAGAGTGCTG GGCAGT-3′, and reverse: 5′-TCAGCAGCTCATGCC CTAAC-3′; GAPDH: forward, 5′-CTGGGCTACACTGA GCACC-3′, and reverse: 5′- AAGTGGTCGTTGAG GGCAATG-3′; miR-145: forward: 5′-GTCCAGTTTTCCC AGGAATCCCT-3′ and reverse: 5′-GCTGTCAACGATA CGCTACCTA-3′; U6 forward: 5′-CGCTTCGGCAGCAC ATATACTA-3′ and reverse: 5 ’-CGCTTCACGAATTT GCGTGTCA-3′; FSCN1: forward: 5′-CCA GGG TAT GGA CCT GTCTG-3′, and reverse: 5′-GTG TGG GTA CGG AAG GCAC-3′.

### Cell transfection

The cDNA encoding linc-ROR was PCR-amplified and subcloned into the pLenti-GIII-CMV-Puro vector (ABM, Canada), which was named as ROR. The sh-RNA specifically targeting linc-ROR were synthesized by ABM (Canada). The sh-RNA sequence for linc-ROR were sh-ROR-1, 5′-GCCTCTGCACTCTTATGG AAGGAGGAAAT-3′, sh-ROR-2, 5′-AGAGTGAAAGTC CCAGGGCATGTGGGAAT-3′, sh-ROR-3, 5′-GGTGAG AAACCCATTGTTCAGTTCCCTAA-3′, sh-ROR-4, 5′-A CTGAGTTGATGATGGAACAGTAGAGTGG-3′.

For FSCN1 functional analysis, cells were transfected with FSCN1-specific small interfering (sh)RNA (ABM, Canada) or pcDNA3.1-FSCN1 plasmids (ABM, Canada). The sh-RNA sequence for FSCN1 were sh-FSCN1-1, 5′-CGAGGACCGCCTGTCCTGCTTCGCGC AGA-3′, sh-FSCN1-2, 5′-GCCGAGAAGTGGAGCG TGCACATCGCCAT-3′, sh-FSCN1-3, 5′-GACAAGGAC GGCAACGTGACCTGCGAGCG-3′, sh-FSCN1-4, 5′-CT GGAGTTCCGCTCCGGCAAGGTGGCCTT-3′.

Transfections were performed using the Lipofectamine 2000 kit (Invitrogen) according to the manufacturer's instructions. Hsa-miRNA-145 mimic/negative control mimic and hsa-miRNA-145 inhibitor/negative control inhibitor were purchased from Applied Biological Materials (ABM, Canada).

### Construction of stable cell lines with overexpression or downregulation of linc-ROR

Cell lines stably expressing or suppressing linc-ROR were constructed by transfecting with lentivirus construct containing desired vector, and screened with Puromycin (2 μg/ml) for four weeks.

### luciferase reporter assay

pmirGLO, pmirGLO-RORwt or pmirGLOROR-mut (miR-145) was co-transfected with miR-145 mimics or miRNA NC into SPC-A1 cells by Lipofectamine-mediated gene transfer. The relative luciferase activity was normalized to Renilla luciferase activity 48 h after transfection. The data were relative to the fold change of the corresponding control groups defined as 1.0.

### *In vitro* chemosensitivity assay

Chemosensitivity was measured by 3-(4, 5-dimethylthiazol-2-yl)-2,5-diphenyl-tetrazolium bromide (MTT, Sigma, USA) assay. Cells were cultured in 96-well plates treated with docetaxel. After 48 h, the MTT solution (5 mg/ml, 20 μl) was added to each well. Following incubation for 4 h, the media was removed and 100 μl DMSO were added to each well. The relative number of surviving cells was assessed by measuring the optical density (O.D.) of cell lysates at 560 nm. All assays were performed in triplicate.

### Colony formation assay

Cells (500 cells/ well) were plated in 6-well plates and incubated in RPMI 1640 with 10% FBS at 37°C. Two weeks later, the cells were fixed and stained with 0.1% crystal violet. The number of visible colonies was counted manually.

### Flow cytometric analysis

Apoptosis assays and cell cycle assays by flow cytometric analysis were described previously. 23 An annexin V-fluorescein isothiocyanate (FITC) apoptosis detection kit and cell cycle detection kit (KeyGEN Biotech, Nanjing, China) was used to inspect apoptosis process and cell cycle distribution in triplicate according to the manufacturer's instructions.

### Cell migration and invasion assays

Cell migration and invasion were measured by transwell chamber (8 um pore size, Corning) and Matrigel invasion (Bection Dickinson), respectively. Forty-eight hour after transfection, cells in serum-free media were placed into the upper chamber coated with or without 10 μg/ml Matrigel. Media containing 10% FBS was added into the lower chamber. Following 48 h incubation, cells remaining in upper membrane were wiped off, while cells that migrated or invaded were fixed in methanol, stained with 0.1% crystal violet and counted under a microscope. Three independent experiments were carried out.

### Immunofluorescence

Cells seeded on glass coverslips in 6-well plates were fixed in 4% formaldehyde solution and permeabilized with 0.5% Triton X-100/PBS. Cells were blocked with 5% BSAPBS for 1 h at room temperature and incubated with primary antibody at 4°C overnight, followed by incubation with fluorescent-dye conjugated secondary antibody (Invitrogen) for 1 h, and then stained with DAPI. Finally, images were taken under an inverted fluorescence microscope.

### Western bolt analysis and antibodies

Total protein lysates were separated in 10% sodium dodecyl sulfate-polyacrylamide gel electrophoresis (SDS-PAGE), and were electrophoretically transferred to polyvinylidene difluoride membranes (Roche). Protein loading was estimated using mouse anti-GAPDH monoclonal antibody. The membranes were blotted with 10% non-fat milk in TBST for 2 h at room temperature, washed and then probed with the rabbit anti-human E-cadherin (1: 2000 dilution), β-catenin (1: 2000 dilution), N-cadherin (1: 2000 dilution), vimentin (1: 2000 dilution), FSCN1 (1:2000 dilution) and GAPDH (1: 3000 dilution), overnight at 4°C, followed by treatment with secondary antibody conjugated to horseradish peroxidase for 2 h at room temperature. The proteins were detected using an enhanced chemiluminescence system and exposed to x-ray film. All antibodies were purchased from Abcam (USA).

### RNA immunoprecipitation (RIP)

RNA immunoprecipitation was performed using thermo fisher RIP kit (Thermo, USA) based on the manufacturer's protocol. The Ago2 antibodies were purchased from Abcam (USA). Normal mouse IgG (Abcam, USA) was applied as negative control and anti- SNRNP70 (Abcam, USA) was employed as positive control for the RIP procedure. Purified RNA was subjected to qRT-PCR analysis to demonstrate the presence of the binding targets using respective primers.

### Xenograft transplantation assays

All female athymic BALB/c nude mice (4–6 week old) were purchased from the Comparative Medicine Department (Jinling Hospital, Nanjing, China). The animal study has been ethically approved and performed according to the institutional guidelines. Subcutaneous xenografts model were established by subcutaneously injecting 2 × 10^6^ SPC-A1/DTX (or H1299/DTX) directly or stable transfected with sh-ROR or sh-control (*n* = 6 mice/group). Tumor growth was under surveillance and tumor volumes were calculated with equation. All mice were sacrificed about 8 weeks later.

Specimens were collected and embedded in paraffin after fixed by 4% paraformaldehyde, and stained with hematoxylin and eosin, as well as immunohistochemistry. All animal experiments were certificated by Jiangsu Province Animal Care and Use Committee.

### TUNEL assay

Apoptosis in transplanted-tumor tissues was detected using the TUNEL assay, performed according to the guidelines recommended by the TUNEL assay kit (KeyGen, Nanjing, China).

### Statistical analysis

Data are shown as the means ± standard error of at least three independent experiments. The SPSS 17.0 software (SPSS Inc., Chicago, IL, USA) was used for statistical analyses. Two group comparisons were performed with a Student *t* test. Multiple group comparisons were analyzed with one-way ANOVA. All tests performed were two-sided.

## SUPPLEMENTARY MATERIALS FIGURES AND TABLES


