# Supplementary material for: Long noncoding RNA ROR regulates chemoresistance in docetaxel-resistant lung adenocarcinoma cells via epithelial mesenchymal transition pathway
Source: Oncotarget. 2017 Mar 25;8(20):33144–58. doi: 10.18632/oncotarget.16562 (PMC5464857; doi:10.18632/oncotarget.16562)
Supplement: Supplementary file 1 [file oncotarget-08-33144-s001.pdf]

Long noncoding RNA ROR regulates chemoresistance in docetaxel-resistant lung adenocarcinoma cells via epithelial mesenchymal transition pathway

Supplementary Materials

Supplemental Table 1: Initial profiling of lncRNAs in docetaxel-resistant cells (SPC-A1/DTX and H1299/DTX) compared with parental cells (SPC-A1 and H1299)

	LncRNAs	fold-change
1	CUST_421_PI428631609	9.972491
2	pl053552	9.575738
3	lnc-ROR	9.053626
4	CUST_2222_PI428631609	8.7036495
5	CUST_6987_PI428631609	8.667568
6	pl011310	8.39463

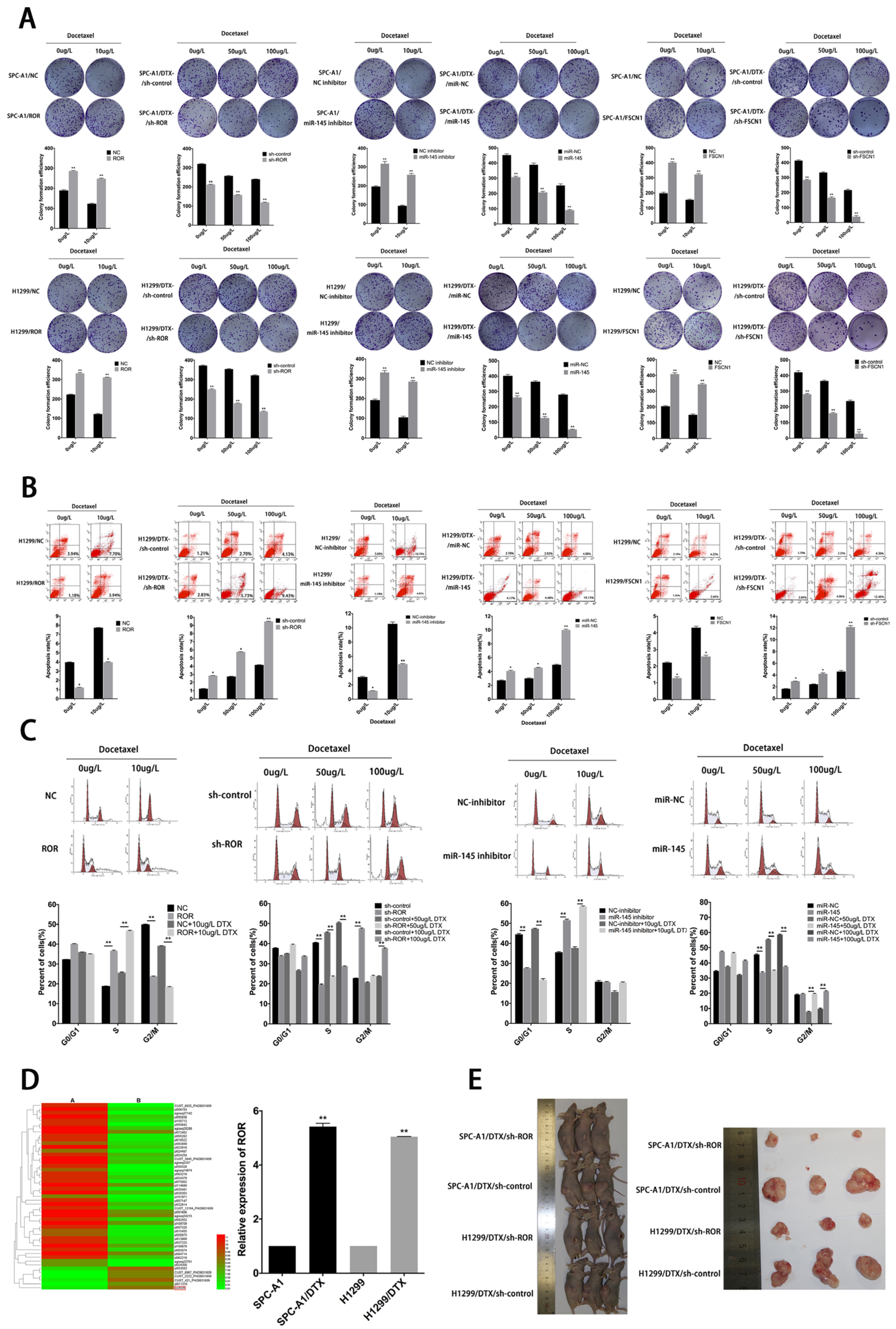

Supplementary Figure 1: (**A**) Colony formation assays to measure cell proliferation in parental cells (SPC-A1 and H1299) transfected with linc-ROR or miR-145 inhibitor or FSCN1, and docetaxel-resistant cells (SPC-A1/DTX and H1299/DTX) transfected with sh-ROR or miR-145 mimics or sh-FSCN1. (**B**, **C**) Flow cytometry analysis to measure apoptosis and cell cycle in H1299 cells transfected with linc-ROR or miR-145 inhibitor or FSCN1, and H1299/DTX cells transfected with sh-ROR or miR-145 mimics or sh-FSCN1. (**D**) Differences lncRNAs expression levels between mixture A (SPC-A1 and H1299) and mixture B (SPC-A1/DTX and H1299/DTX). The expression level of linc-ROR was significantly upregulated in mixture B (9.05-fold). (**E**) Expression level of linc-ROR in parental and docetaxel-resistant LAD cell lines. Data are presented as mean ± standard error based on at least three independent experiments. *$p < 0.05$, **$p < 0.01$.

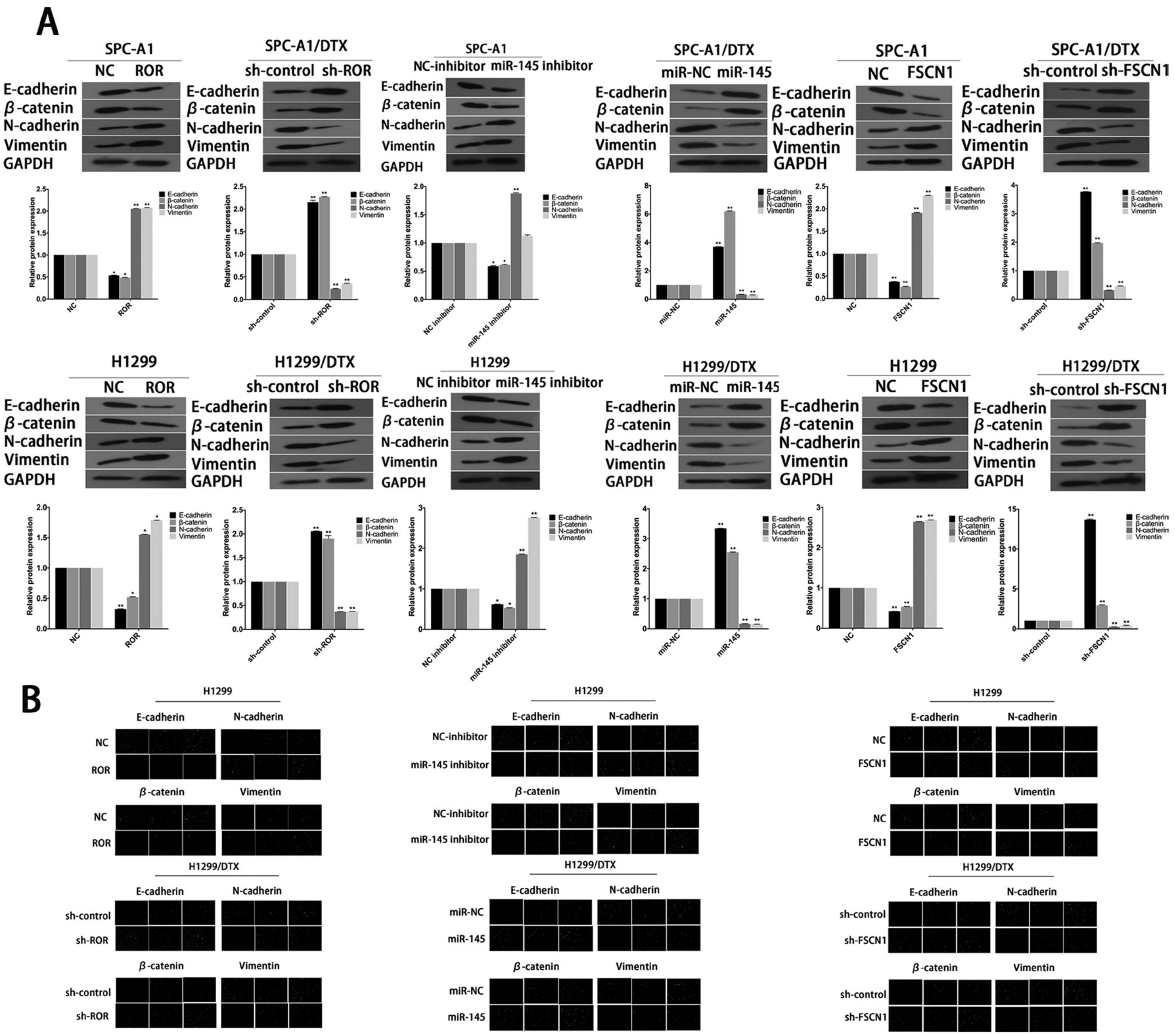

Supplementary Figure 2: (**A**) Western blotting analysis of the expression of epithelial markers (E-cadherin, β-catenin) and mesenchymal markers (N-cadherin, vimentin) in parental cells transfected with linc-ROR or miR-145 inhibitor or FSCN1, and docetaxel-resistant cells transfected with sh-ROR or miR-145 mimics or sh-FSCN1. (**B**) Immunofluorescence analysis of the change in epithelial and mesenchymal markers in H1299 cells transfected with linc-ROR or miR-145 inhibitor or FSCN1, and H1299/DTX cells transfected with sh-ROR or miR-145 mimics or sh-FSCN1.

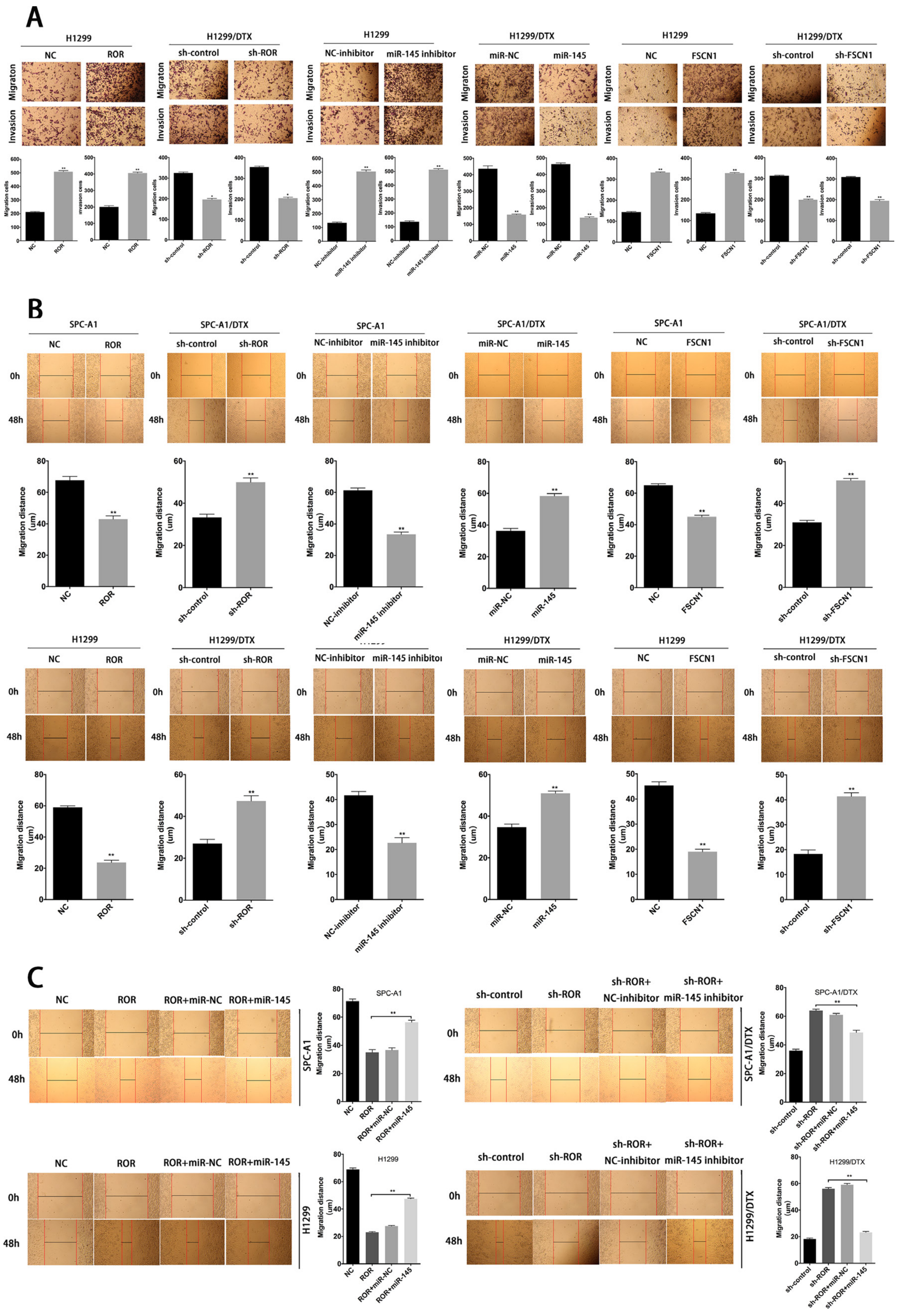

Supplementary Figure 3: (**A**) Transwell assays to determine cell migration and invasion ability of H1299 cells transfected with linc-ROR or miR-145 inhibitor or FSCN1, and H1299/DTX cells transfected with sh-ROR or miR-145 mimics or sh-FSCN1. (**B**) Wound healing assays to determine cell migration and invasion ability of parental cells transfected with linc-ROR or miR-145 inhibitor or FSCN1, and docetaxel-resistant cells transfected with sh-ROR or miR-145 mimics or sh-FSCN1. (**C**) Metastasis capacity of parental cells stably transfected with linc-ROR and docetaxel-resistant cells stably transfected with sh-ROR was partially reversed by miR-145 mimics and miR-145 inhibitor, respectively.
